# Residual HEMA and TEGDMA Release and Cytotoxicity Evaluation of Resin-Modified Glass Ionomer Cement and Compomers Cured with Different Light Sources

**DOI:** 10.1155/2014/218295

**Published:** 2014-01-28

**Authors:** Murat Selim Botsali, Adem Kuşgöz, Subutay Han Altintaş, Hayriye Esra Ülker, Mehmet Tanriver, Serdar Kiliç, Feridun Başak, Mustafa Ülker

**Affiliations:** ^1^Department of Pedodontics, Faculty of Dentistry, Selcuk University, Selcuklu 42079, Konya, Turkey; ^2^Department of Pedodontics, Faculty of Dentistry, Karadeniz Technical University, 61080 Trabzon, Turkey; ^3^Department of Prosthodontics, Faculty of Dentistry, Karadeniz Technical University, 61080 Trabzon, Turkey; ^4^Department of Restorative Dentistry, Faculty of Dentistry, Selcuk University, 42079 Konya, Turkey; ^5^Department of Pedodontics, Faculty of Dentistry, Şifa University, 35535 İzmir, Turkey; ^6^Department of Pedodontics, Centre for Dental Sciences, Gulhane Military Medical Academy, 06010 Ankara, Turkey

## Abstract

The purpose of this study was first to evaluate the elution of 2-hydroxyethyl methacrylate (HEMA) and triethylene glycol dimethacrylate (TEGDMA) monomers from resin-modified glass ionomer cement (RMGIC) and compomers cured with halogen and light-emitting diode (LED) light-curing units (LCUs). The effect of cured materials on the viability of L929 fibroblast cells was also evaluated. One RMGIC (Ketac N100) and two compomers (Dyract Extra and Twinkystar) were tested. Materials were prepared in teflon disks and light-cured with LED or halogen LCUs. The residual monomers of resin materials in solution were identified using high-performance liquid chromatography. The fibroblast cells' viability was analyzed using MTT assay. The type of LCU did not have a significant effect on the elution of HEMA and TEGDMA. A greater amount of HEMA than TEGMDA was eluted. The amount of TEGDMA eluted from Twinkystar was greater than Dyract Extra (*P* < 0.05) when cured with a halogen LCU. All material-LCU combinations decreased the fibroblast cells' viability more than the control group (*P* < 0.01), except for Dyract Extra cured with a halogen LCU (*P* > 0.05). Curing with the LED LCU decreased the cells' viability more than curing with the halogen LCU for compomers. For Ketac N100, the halogen LCU decreased the cells' viability more than the LED LCU.

## 1. Introduction

Resin-modified glass ionomer cements (RMGICs) and compomers play an important role in permanent and primary dentition with their specific conditions. Compomers were developed with the aim of combining the positive properties of light-cured composites with those of glass ionomer cements. RMGICs are characterized by their improved physical and mechanical properties in comparison with conventional glass ionomer cements (GICs). Due to these advantages and their ease of application, providing good aesthetics, bonding to dental hard tissue, fluoride release, and radiopacity, RMGICs and compomers are considered a useful alternative to amalgam in restorative and pediatric dentistry [1, 2].

Generally, a complete conversion from monomers to polymers is not possible [3, 4]. Incomplete polymerization of resin-based restorative material and the leaching of monomers not only decrease the mechanical properties of a restoration, but can also negatively impact the material's biocompatibility [5–7]. Liquids can leach these intact components from a restoration toward the pulp or oral environment. There is also a correlation between the amount of uncured resin monomers in a restorative material and the magnitude of the cytotoxicity effect [6–8].

RMGICs and compomers can have local and systemic adverse effects. These effects can be caused by substances that are released from resinous materials after polymerization [6, 9–11]. Studies on the degradation of dental materials have confirmed the release of substances such as 2-hydroxyethyl methacrylate (HEMA) and triethylene glycol dimethacrylate (TEGDMA) from resin-based dental materials [6–9, 12, 13]. HEMA and TEGDMA are a likely cause of cellular stress via the formation of reactive oxygen species (ROS). Demirci et al. found a possible link between ROS production and cytotoxic activity [14]. Moreover, the induction of genotoxic effects of TEGDMA and HEMA has been demonstrated *in vitro* as well, indicating the compounds' DNA reactivity [15]. In addition, cytotoxic resin materials have been shown to cause cytotoxicity and elevated numbers of micronuclei [16]. Geurtsen et al. showed that the elution of TEGDMA was one of the main causes of the cytotoxic reactions evoked by the light-cured glass ionomer cements and compomers they investigated [6]. Thus, this comonomer's liberation from resin restorations should be minimized or prevented [6].

For many years, halogen light-curing units (LCUs) were preferred as the most practical method for polymerizing light-cured resin. However, as halogen LCUs exhibit several shortcomings, as an alternative, a light-emitting diode (LED) LCU was introduced to polymerize light-cured resin. However, conflicting results have often been observed in the literature related to the effects of both LCUs. Some authors have claimed that the curing performance of second-generation LED LCUs is similar to or better than that of halogen LCUs [17–19]. In contrast, others have reported that the curing performance of halogen LCUs was better than that of LED LCUs [20, 21]. A few studies have focused on the components that are leached from RMGICs and compomers that are cured using these LCUs [6]. Although much literature has been published on the release of monomers from composite materials [7, 12, 13, 22], information is still lacking with respect to the elution and cytotoxicity of monomers from modern RMGICs and compomer materials that are used daily in clinical practice. Therefore, new studies are necessary to evaluate these materials under *in vitro* conditions.

Regarding resin monomers' importance in polymer conversion in RMGICs and compomers and the uncured soluble components' toxic effects in a moist environment, the purposes of this *in vitro* study are as follows: (1) to evaluate the elution of residual monomers (HEMA and TEGDMA) from the RMGIC and compomers when cured with halogen and LED LCUs and (2) to investigate the effect of RMGICs and compomers cured with different LCUs (halogen and LED) on fibroblasts' viability.

## 2. Materials and Methods

The commercially available RMGIC (Ketac N100, 3 M ESPE, USA) and compomers (Dyract Extra, Dentsply, USA, and Twinkystar, Voco, Germany) tested in this study are listed in Table 1.

### 2.1. High-Performance Liquid Chromatography (HPLC) Analysis

A total of 20 disk-shaped specimens 5 mm in diameter and 2.5 mm in thickness were prepared from each material using a teflon mold. The molds were filled with uncured material and covered with a mylar strip to protect the resin cement from the oxygen inhibition zone, and materials were polymerized by LCUs. A halogen LCU (Astralis 3, Ivoclar Vivadent, Schaan, Liechtenstein) with an output irradiance of 530 mW/cm^2^ and an LED LCU (Elipar FreeLight 2, 3M ESPE, St. Paul, MN, USA) with an output irradiance of 1000 mW/cm^2^ were used for curing. The exposure times were chosen for halogen (40 s) and LED LCUs (20 s) based on the manufacturers' recommendations. During specimen preparation, irradiance was periodically checked with a dental radiometer (Curing Radiometer, HILUX/Benlioglu Corp., Ankara, Turkey).

Cured samples were detached from the teflon molds and immediately immersed in light-proof glass bottles containing 1.5 mL of 75% ethanol and 25% deionized water after resin cements were polymerized; samples were stored at 37°C for four minutes and 24 hours. To avoid contamination from other polymer-based materials and plastics, gloves were not used. The extracts were taken off from bottles after 4 minutes and 24 hours. The light-proof glass bottles were distilled with ethyl acetate twice and kept at 100°C for at least 12 hours before use.

All measurements were performed three times for each of the extracts. Calibration curves were made relating the eluted peak area to known concentrations of TEGDMA and HEMA. Standard chromatograms of TEGDMA and HEMA were obtained. The concentrations of the leaching monomers from tested restorative resins for per-storage periods were calculated using the coefficients obtained from a linear regression analysis of the results from the standard series.

The residual monomers of resin material in solution were identified using high-performance liquid chromatography with ultraviolet detection. Identification was confirmed with reference substances.

The analysis of extracts from the resin material as well as reference solutions of the monomers in water/acetonitrile (25 : 75%) was carried out using HPLC (Agilent Technologies, USA) with the following conditions:

- column: steel column (Waters Corporation, Milford Massachusetts, USA), 250 mm in length, 4.6 mm in diameter, and a particle size of 5 **μ**M;
- mobile phase: CH_3_CN 75%/H_2_O 25% (Acetonitrile);
- flow speed: 1 mL/min.;
- detection: UV: 208 nm for TEGDMA and HEMA;
- injection: 10 **μ**L loop at constant room temperature.

### 2.2. Cytotoxicity Testing

Test specimens were prepared according to the manufacturers' instructions in standard teflon disks (5 mm in diameter, 2.5 mm high) and light-cured with LED or halogen LCUs. All specimens were prepared and handled under aseptic conditions to limit the influence of biological contamination on the cell culture tests. Extracts of these samples were prepared following the recommendations of ISO 10993-12 at a ratio of 117.8 mm^2^ sample surface area/mL cell culture medium. In detail, 15 samples were extracted in 5 mL of cell culture medium for 24 hours at 37°C and 5% CO_2_. The culture medium containing material extracts was sterilely filtered to use on the cell cultures.

The L929 fibroblast cell line (ATCC CCL 1) was cultured in Basal Medium Eagle (BME) (Biological Industries, Beit Haemek, Israel) containing 10% of newborn calf serum (Biological Industries, Beit Haemek, Israel) and 100 mg/mL of penicillin/streptomycin (Biological Industries, Beit Haemek, Israel) at 37°C and 5% CO_2_. Confluent cells were detached with 0.25% trypsin and seeded in 96-well plates (25.000 cells/mL). After 24 hours of incubation, the culture medium was replaced with 200 *μ*L of culture medium containing material extracts of tested materials. The original culture medium served as the control in this study. Cultures were incubated at 37°C and 5% CO_2_ for 24 hours. The viability of cells exposed to material extracts was assessed using their succinic dehydrogenase activity. Succinic dehydrogenase activity has been shown to be reasonably representative of mitochondrial activity in cells and reflects both cells' number and activity. The old medium was removed and cell cultures were rinsed with phosphate buffer saline (PBS); 200 *μ*L aliquots of freshly prepared MTT [3-(4,5-dimethyl-thiazol-2-yl)-2,5-diphenyl-tetrazolium bromide, Sigma Aldrich, Germany] solution (0.5 mg/mL in BME) were added to each well. After two hours of incubation (37°C, 5% CO_2_), the supernatant was removed and the intracellularly stored MTT formazan was solubilized in 200 *μ*L of dimethyl sulfoxide for 30 minutes at room temperature. The absorbance at 540 nm was spectrophotometrically measured. Twelve replicate cell cultures were exposed to extracts of the materials (*n* = 12). The cell survival of treated groups was compared to that of untreated controls.

### 2.3. Statistical Analyses

Statistical analyses were performed with SPSS for 13.0 Windows (SPSS Inc, Chicago, IL, USA). The data on residual monomers eluted from resin were analyzed by two-way ANOVA using Tukey's HSD post hoc analysis at a significance level of *P* < 0.05. The data on cell viability were also analysed by two-way ANOVA, and as a result, an interaction between two factors (tested materials and LCUs) was observed. Because the data did not show a normal distribution, a significant difference was evaluated using the Kruskal-Wallis test and the Bonferoni-corrected Mann-Whitney *U* test.

## 3. Results

### 3.1. Monomer Elution

The mean values and the standard deviations of the monomers released from the two resin composites for the different storage times and different LCUs are depicted in Table 2. The two-way ANOVA indicated that for the four-minute and 24-hour periods, the type of LCU did not have a significant effect on the elution of HEMA and TEGDMA. Regardless of the LCU, the kind of material, and the storage time, more HEMA than TEGDMA was eluted from the three tested materials.

However, amount of residual monomer values varies according to the materials tested (Table 2). The amount of eluted TEGDMA from Twinkystar was greater than Ketac N100 (*P* < 0.05), and the amount of eluted TEGDMA from Ketac N100 was greater than Dyract Extra (*P* < 0.05) when cured with the halogen LCU and measured after four minutes. The amount of eluted TEGDMA from Twinkystar was greater than Dyract Extra (*P* < 0.05) when cured with the halogen LCU and measured after 24 hours. Similar amounts of TEGDMA were released from tested materials when cured with the LED LCU (*P* < 0.05). Similar amounts of HEMA were released from tested materials when cured with both halogen and LED LCUs (*P* > 0.05).

### 3.2. Cytotoxicity

**Figure 1:**
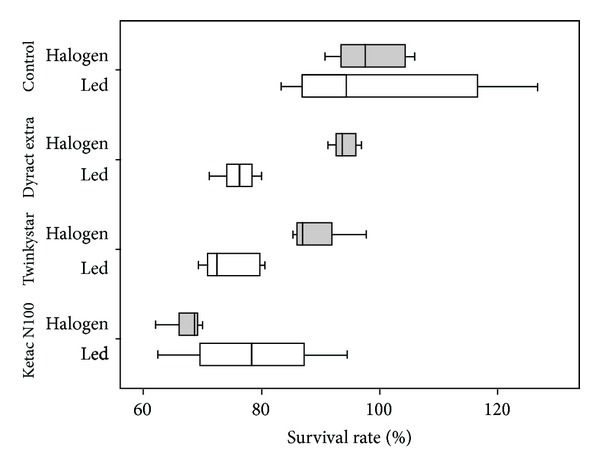
Cytotoxicity of resin-modified glass inomer cement and compomers cured with halogen and LED light curing units. Data are expressed as percentage of the negative control cultures (*n* = 12).

The results of cytotoxicity testing are shown in Figure 1. The results show that, when cured with the LED LCU, all materials tested decreased the fibroblast cells' viability when compared with the control group (*P* < 0.01). All tested materials decreased cells' viability similarly (*P* > 0.05). When cured with the halogen LCU, the cell viability of Dyract Extra did not differ from that of the control group (*P* > 0.05), but Twinkystar and Ketac N100 decreased the cells' viability compared with control group (*P* < 0.01). Ketac N100 decreased the cells' viability more than Twinkystar, and Twinkystar decreased the cells' viability more than Dyract Extra (*P* < 0.05). Curing with the LED LCU decreased the cells' viability more than curing with the halogen LCU for both compomers (Dyract Extra and Twinkystar); *P* = 0.000 and *P* = 0.008, respectively. For Ketac N 100, the halogen LCU decreased the cells' viability more than the LED LCU (*P* = 0.011).

## 4. Discussion

The quality and quantity of the residual monomers eluted from dental resin materials are usually determined using high-performance liquid chromatography (HPLC) [23, 24] since it is a very powerful and commonly used separation method. It is preferred to gas chromatography as it gives a greater level of control over the separation process since monomers are soluble in the mobile phase [25, 26]. HPLC analysis was therefore used in this study to evaluate monomers' release from tested restorative resin materials.

Degradation of resins in the oral cavity depends on salivary enzymatic reactions, acidic conditions, and erosive factors caused by foods and drinks [27]. Organic solvents such as ethanol, methanol, or mixtures of these solvents with water are preferred to simulate oral conditions [26, 28]. Organic solvents have the ability to penetrate and swell the polymer network, facilitating the liberation of unreacted and leachable monomers. As a solvent penetrates the matrix and expands the openings between polymer chains, oligomers diffuse out [26, 28]. The intraoral fluids fall somewhere in between the more aggressive organic solvents and water, and the US FDA recommends the 75% ethanol-water solution as a food/oral simulating liquid as clinically relevant [29]. Therefore, in the present study, 75% ethanol-25% deionized water was used as the extraction media to measure the monomers' release.

According to national and international regulations, dental materials have to be evaluated for biocompatibility before being applied to patients. For this purpose, animal experiments and cell culture tests are available. Animal experiments to test the cytotoxicity of dental materials are time consuming, expensive, and subject to extensive public discussions. *In vitro* cytotoxicity testing has the advantage of allowing for easy control of experimental factors that are often problematic when performing experiments *in vivo*. *In vitro* methods are reproducible, cost-effective, relevant, and suitable for the evaluation of basic biological properties of dental materials [30, 31]. Different *in vitro* testing methods and cell lines have been used to determine dental materials' cytotoxicity. In the present study, the effect of RMGICs and compomers on fibroblast cells was investigated using the MTT test. Fibroblasts are the targets of any chemical components released from dental restorative materials. L929 fibroblast cells were selected due to their availability, popularity, and efficiency to grow *in vitro* [8, 32]. MTT assay is a well-established method for analyzing cell viability [8, 31, 33]. The viability and proliferation of cells are assessed by means of the functional state of cells' mitochondria. Mitochondrial dehydrogenases in living cells reduce the yellow tetrazolium salt, MTT, to blue MTT formazan, which is then retained in the cells. The formation of the formazan product has been found to correlate well with the number of viable cells.

A greater amount of HEMA was released from the tested materials than TEGDMA. An important factor affecting residual monomer release is the chemistry and size of the monomers in the resin materials. Smaller molecules are expected to leach more and faster than larger molecules. The molecular weights of HEMA and TEGDMA are 130.14 g/moL and 236.33 g/moL, respectively. HEMA is listed as an ingredient by the producers of RMGICs; however, it is not listed as an ingredient by the producers of compomers, but Geurtsen et al. have confirmed its presence in compomers [6]. HEMA may be a degradation product from urethane dimethacrylate (UDMA) [34], which is an ingredient in both Dyract Extra and Twinkystar, according to material safety data sheets.

The two types of light sources evaluated in this study did not differ statistically in terms of the residual amounts of monomers released from tested restorative materials at two different time periods. Yap et al. reported that monomer release was higher in LED devices than in halogen devices in their study in which they investigated the effects of five different light devices on residual monomer release from composite materials with an HPLC device [35]. We consider that the residual monomer amount released in LED groups is higher than in halogen groups because the wavelength interval of the light generated by LED light sources has a tighter spectral distribution than halogen light sources. The fact that there is no significant difference in terms of the monomer release values between the light sources used in the scope of our study suggests that when these devices are used in ideal conditions, their polymerization capacities can approximate one another. On the other hand, the results of cytotoxicity testing revealed that the type of LCU may affect the toxic potential of compomers and RMGICs and that this effect is material dependent. The determination of a possibly toxic effect of the resin-based restorative materials is a matter of interest. In view of the great variety of LCUs and filling materials currently in use, the question is which combinations cause the least toxic effects [36]. Compomers are more toxic when they are polymerized with LED LCUs; however, RMGICs are more toxic when polymerized with halogen LCUs. In accordance with our results, Tunç et al. reported that compomers are potentially toxic to human pulp fibroblasts and LED LCUs may not be appropriate for use in the polymerization of compomers because compomer specimens showed greater decolorization when cured with LED than with halogen LCUs [11]. Similarly, Yap et al. found LED-cured composite to be more cytotoxic than composite cured with conventional halogen light [35]. The fact that compomers contain both camphorquinone photoinitiators and other coinitiators may be the reason for the higher cytotoxicity found in compomer specimens cured with LED LCUs compared to those cured with halogen LCUs [6]. It has also been reported that light activation plays an important role in reducing the cytotoxicity of RMGIC [37]. Output irradiance of LED LCU used (1000 mW/cm^2^) is much higher than the output irradiance of halogen LCU (530 mW/cm^2^) used. This may explain the lower cytotoxicity found in RMGIC specimens cured with the LED LCU than in those cured with the halogen LCU in this study.

Few reports have been published about the eluted residual monomers of RMGIC and compomers. This study evaluated the HEMA and TEGDMA eluted from RMGIC and compomers. HEMA elution was similar for RMGICs and compomers; however, TEGDMA elution from the Twinkystar compomer was higher than the other compomer tested (Dyract Extra) and the RMGIC (Ketac N100). Several factors contribute to the process of elution from resin-based dental materials. According to Ferracane, the first relates to the amount of components released, and this should be directly related to the extent of the polymerization reaction (i.e., the degree of double bond conversion) [29]. Second, the solvents' chemistry significantly affects elution. Third, the size and the chemical nature of the released components play a role. Additionally, the composition (filler loading) of the resin-based dental materials directly influences the elution of monomers [38]. It is more likely that the difference in the chemistry of the three materials, the filler size, and the distribution of the filler particles could have influenced our results [38]. On the other hand, the cytotoxicity data of tested materials are not fully in accordance with the monomer releasing data. Ketac N100 was determined to be the most cytotoxic material; however, the least amount of TEGDMA elution was obtained with that material. In other words, RMGIC is more cytotoxic than the compomers. In the light curing of RMGICs, rapid polymerization occurs, associated with a slow acid-base reaction and resulting in a prolonged period of acid release. This is responsible for the maintenance of lower pH values for extended lengths of time, which may contribute to the material's higher cytotoxicity [37, 39].

The acid-base reaction in RMGICs is extended due to the fact that HEMA, presenting a hydrophilic group in its composition, absorbs water during the polymerization process, which is essential to the chemical reaction [37, 40]. In addition, fluoride release might also contribute to the cytotoxic effects. A recent study demonstrated that low levels of released fluoride correlated to low cytotoxic effect of fluoride releasing materials [41].

## 5. Conclusion

The findings presented here imply that biologically active substances such as HEMA and TEGDMA may be released from RMGICs and compomers and eventually damage fibroblast cells. The toxic potential of RMGICs and compomers may vary depending on the type of LCU used for polymerization.

## Figures and Tables

**Table 1:** Composition of the resin modified glass ionomer cement and compomers used in this study.

Materials	Organic matrix	Inorganic filler		
		(Filler content)		Size
Dyract Extra Lot#0809000332 Dentsply, USA	UDMA, carboxylic acid modified dimethacrylate resin, TEGDMA, BHT, strontium alimino-sodium-fluoro-silicate glass	Strontium fluoride glass particles	50% volume	0.8 *μ*m

Twinkystar Lot#0919531 Voco, Germany	BisGMA, UDMA, TEGDMA, carboxylicacid modified methacrylate, camphorquinone, BHT	Barium aluminium fluorosilicate glass, strontium fluorosilicate glass, silicon dioxide	77.8% w/w	0.4–3.0 *μ*m

Ketac N100 Lot#20071015 3M ESPE, Germany	Blend including HEMA, TEGDMA, methacrylate modified polyalkenoic acid	Fluoroaluminosilicate glass, Nanomers, nanoclusters	69% by weight	5–25 nm and 1.0 to 1.6 *μ*m

**Table 2:** The residual monomer concentrations eluted from resin modified glass ionomer cement and compomers cured with halogen and LED light curing units in different time periods (mean ± sd).

Time	Monomers	Light curing unit	Materials			
			Dyract Extra	Twinkystar	Ketac N100	*P *
4 minutes	HEMA	Halogen	8.3 ± 1.2	7.4 ± 1.8	8.5 ± 2.6	ns
		LED	9.9 ± 1.2	7.3 ± 1.0	7.1 ± 2.2	ns
	TEGDMA	Halogen	0.4 ± 0.05	2.4 ± 0.5^a^	1.07 ± 0.8^a,b^	<0.05
		LED	1.0 ± 1.5	2.6 ± 1.1	1.2 ± 0.6	ns

24 hours	HEMA	Halogen	20.4 ± 4.5	14.6 ± 5.4	18.5 ± 4.7	ns
		LED	20.1 ± 2.9	16.7 ± 1.6	16.8 ± 3.1	ns
	TEGDMA	Halogen	1.1 ± 0.1	6.8 ± 4.8^a^	4.5 ± 2.6	<0.05
		LED	2.4 ± 2.2	6.5 ± 3.0	4.3 ± 2.1	ns

^a^ *P* < 0.05 compared to Dyract extra.

^b^ *P* < 0.05 compared to Twinkystar.

The concentration values were calculated as *μ*M (micromolarity).

## References

[1] Qvist V, Poulsen A, Teglers PT, Mjör IA (2010). The longevity of different restorations in primary teeth. *International Journal of Paediatric Dentistry*.

[2] Forss H, Widström E (2004). Reasons for restorative therapy and the longevity of restorations in adults. *Acta Odontologica Scandinavica*.

[3] Asmussen E (1982). Factors affecting the quantity of remaining double bonds in restorative resin polymers. *Scandinavian Journal of Dental Research*.

[4] Imazato S, McCabe JF, Tarumi H, Ehara A, Ebisu S (2001). Degree of conversion of composites measured by DTA and FTIR. *Dental Materials*.

[5] Munksgaard EC, Freund M (1990). Enzymatic hydrolysis of (di)methacrylates and their polymers. *Scandinavian Journal of Dental Research*.

[6] Geurtsen W, Spahl W, Leyhausen G (1998). Residual monomer/additive release and variability in cytotoxicity of light-curing glass-ionomer cements and compomers. *Journal of Dental Research*.

[7] Gupta SK, Saxena P, Pant VA, Pant AB (2012). Release and toxicity of dental resin composite. *Toxicology International*.

[8] Kaga M, Noda M, Ferracane JL, Nakamura W, Oguchi H, Sano H (2001). The in vitro cytotoxicity of eluates from dentin bonding resins and their effect on tyrosine phosphorylation of L929 cells. *Dental Materials*.

[9] Goldberg M (2008). In vitro and in vivo studies on the toxicity of dental resin components: a review. *Clinical Oral Investigations*.

[10] Geurtsen W (2000). Biocompatibility of resin-modified filling materials. *Critical Reviews in Oral Biology and Medicine*.

[11] Tunç ES, Özer L, Sari S, Çetiner S (2009). Cytotoxic effects of halogen- and light-emitting diode-cured compomers on human pulp fibroblasts. *International Journal of Paediatric Dentistry*.

[12] van Landuyt KL, Nawrot T, Geebelen B (2011). How much do resin-based dental materials release? A meta-analytical approach. *Dental Materials*.

[13] Mazzaoui SA, Burrow MF, Tyas MJ, Rooney FR, Capon RJ (2002). Long-term quantification of the release of monomers from dental resin composites and a resin-modified glass ionomer cement. *Journal of Biomedical Materials Research*.

[14] Demirci M, Hiller K, Bosl C, Galler K, Schmalz G, Schweikl H (2008). The induction of oxidative stress, cytotoxicity, and genotoxicity by dental adhesives. *Dental Materials*.

[15] Schweikl H, Hiller K, Bolay C (2005). Cytotoxic and mutagenic effects of dental composite materials. *Biomaterials*.

[16] Schweikl H, Schmalz G, Spruss T (2001). The induction of micronuclei in vitro by unpolymerized resin monomers. *Journal of Dental Research*.

[17] Bala O, Ölmez A, Kalayci S (2005). Effect of LED and halogen light curing on polymerization of resin-based composites. *Journal of Oral Rehabilitation*.

[18] Price RBT, Felix CA, Andreou P (2005). Knoop hardness of ten resin composites irradiated with high-power LED and quartz-tungsten-halogen lights. *Biomaterials*.

[19] Ritter AV, Cavalcante LM, Swift EJ, Thompson JY, Pimenta LA (2006). Effect of light-curing method on marginal adaptation, microleakage, and microhardness of composite restorations. *Journal of Biomedical Materials Research B*.

[20] Dunn WJ, Bush AC (2002). A comparison of polymerization by light-emitting diode and halogen-based light-curing units. *Journal of the American Dental Association*.

[21] Beun S, Glorieux T, Devaux J, Vreven J, Leloup G (2007). Characterization of nanofilled compared to universal and microfilled composites. *Dental Materials*.

[22] Polydorou O, König A, Hellwig E, Kümmerer K (2009). Long-term release of monomers from modern dental-composite materials. *European Journal of Oral Sciences*.

[23] Munksgaard EC, Peutzfeldt A, Asmussen E (2000). Elution of TEGDMA and BisGMA from a resin and a resin composite cured with halogen or plasma light. *European Journal of Oral Sciences*.

[24] Altintas SH, Usumez A (2012). Evaluation of TEGDMA leaching from four resin cements by HPLC. *European Journal of Dentistry*.

[25] Moharamzadeh K, van Noort R, Brook IM, Scutt AM (2007). HPLC analysis of components released from dental composites with different resin compositions using different extraction media. *Journal of Materials Science: Materials in Medicine*.

[26] Altintas SH, Usumez A (2008). Evaluation of monomer leaching from a dual cured resin cement. *Journal of Biomedical Materials Research B*.

[27] Eliades T, Eliades G, Brantley WA, Johnston WM (1995). Residual monomer leaching from chemically cured and visible light-cured orthodontic adhesives. *The American Journal of Orthodontics and Dentofacial Orthopedics*.

[28] Komurcuoglu E, Olmez S, Vural N (2005). Evaluation of residual monomer elimination methods in three different fissure sealants in vitro. *Journal of Oral Rehabilitation*.

[29] Ferracane JL (1994). Elution of leachable components from composites. *Journal of Oral Rehabilitation*.

[30] Schmalz G (1994). Use of cell cultures for toxicity testing of dental materials-advantages and limitations. *Journal of Dentistry*.

[31] Kong N, Jiang T, Zhou Z, Fu J (2009). Cytotoxicity of polymerized resin cements on human dental pulp cells in vitro. *Dental Materials*.

[32] Saw TY, Cao T, Yap AUJ, Ng MML (2005). Tooth slice organ culture and established cell line culture models for cytotoxicity assessment of dental materials. *Toxicology In Vitro*.

[33] Wataha JC, Craig RG, Hanks CT (1992). Precision of and new methods for testing in vitro alloy cytotoxicity. *Dental Materials*.

[34] Michelsen VB, Moe G, Skålevik R, Jensen E, Lygre H (2007). Quantification of organic eluates from polymerized resin-based dental restorative materials by use of GC/MS. *Journal of Chromatography B*.

[35] Yap AUJ, Saw TY, Cao T, Ng MML (2004). Composite cure and pulp-cell cytotoxicity associated with LED curing lights. *Operative Dentistry*.

[36] Sigusch BW, Völpel A, Braun I, Uhl A, Jandt KD (2007). Influence of different light curing units on the cytotoxicity of various dental composites. *Dental Materials*.

[37] Aranha AMF, Giro EMA, Souza PPC, Hebling J, de Souza Costa CA (2006). Effect of curing regime on the cytotoxicity of resin-modified glass-ionomer lining cements applied to an odontoblast-cell line. *Dental Materials*.

[38] Polydorou O, Hammad M, König A, Hellwig E, Kümmerer K (2009). Release of monomers from different core build-up materials. *Dental Materials*.

[39] Woolford MJ, Chadwick RG (1992). Surface pH of resin-modified glass polyalkenoate (ionomer) cements. *Journal of Dentistry*.

[40] Wan AC, Yap AU, Hastings GW (1999). Acid-base complex reactions in resin-modified and conventional glass ionomer cements. *Journal of Biomedical Materials Research*.

[41] Kanjevac T, Milovanovic M, Volarevic V (2012). Cytotoxic effects of glass ionomer cements on human dental pulp stem cells correlate with fluoride release. *Medicinal Chemistry*.

